# Successful Management of Periprocedural Coronary Extravasation Using Liquid Embolic Agent n-Hexyl-Cyanoacrylate

**DOI:** 10.3390/jcdd11110347

**Published:** 2024-11-01

**Authors:** Artiomas Širvys, Andrius Berūkštis

**Affiliations:** 1 Faculty of Medicine, Clinic of Cardiac and Vascular Diseases, Vilnius University, 01513 Vilnius, Lithuania; andrius.berukstis@santa.lt; 2 Vilnius University Hospital Santaros Klinikos, 08661 Vilnius, Lithuania

**Keywords:** acute coronary syndrome, percutaneous coronary intervention complication, coronary artery perforation, intracoronary glue

## Abstract

Although the complication rate of percutaneous coronary intervention is low, coronary artery perforation occurs in 0.2–0.5% of cases. Intracoronary glue injection is not an established treatment option, with only a few cases reported in the literature and no reported use of n-hexyl-cyanoacrylate. Case report: A 75-year-old man was diagnosed with a non-ST elevation myocardial infarction. Since there was no acute chest pain and no signs of ongoing ischemia on the ECG, diagnostic coronary angiography was performed the day after arrival. The coronary angiography revealed a proximal subocclusion of the left anterior descending artery. The lesion was successfully predilated, and a drug-eluting 5 × 28 mm stent was implanted, occluding two small diagonal branches. While attempting to create a gap in the stent to revascularize the occluded branch, a side branch perforation was detected. This was successfully treated by occluding the branch with an intracoronary cyanoacrylate glue injection. No signs of cardiac tamponade were observed during follow-up after the procedure, and the patient was soon discharged to rehabilitation. Conclusions: Coronary artery perforation is a serious complication of percutaneous coronary intervention. Intracoronary glue injection and embolization of the perforated side branch appear to be a safe and effective technique for managing this complication.

## 1. Introduction

Since the first percutaneous coronary intervention (PCI) in 1977 by Andreas Gruentzig [1], the procedure technique continues to evolve. There is no debate that PCI improves both the short- and long-term survival of patients with ACS, significantly reducing both mortality and long-term morbidity [2]. Although the complication rate of PCI has decreased over time due to advances in the technique [3,4], complications still occur, with the rate highly dependent on the procedure’s complexity [5]. Coronary artery perforation remains the most feared complication of PCI, representing a nightmare for interventional cardiologists and a potentially life-threatening event for patients if not treated promptly [6]. Luckily, this complication is rare, occurring in 0.2–0.5% of PCI cases in well-experienced centers [7,8]. Cyanoacrylate glue is commonly used in peripheral sites during interventional radiology procedures [9]. However, routine treatments for coronary artery perforation typically do not involve intracoronary glue application [10]. There are only a few case reports in the literature describing the use of intracoronary glue for treating coronary artery perforation [11]. Although this technique is not yet established, the reported cases suggest it may be safe.

This case report presents a rare complication of coronary artery side branch perforation and its treatment with glue injection.

## 2. Case Report

A 75-year-old man with no history of cardiovascular events experienced sudden epigastric pain at rest in the evening, which radiated to his chest. The pain persisted throughout the night, but the patient did not call an ambulance, assuming it was recurring gastric pain due to a previously diagnosed stomach ulcer. The pain subsided on its own over the next few days, leaving him with general weakness and new-onset shortness of breath during mild physical activity. Ten days after the initial chest pain, the patient consulted a cardiologist at a primary care clinic, where an acute myocardial infarction (MI) was suspected, and he was referred to a PCI center.

**Figure 1:**
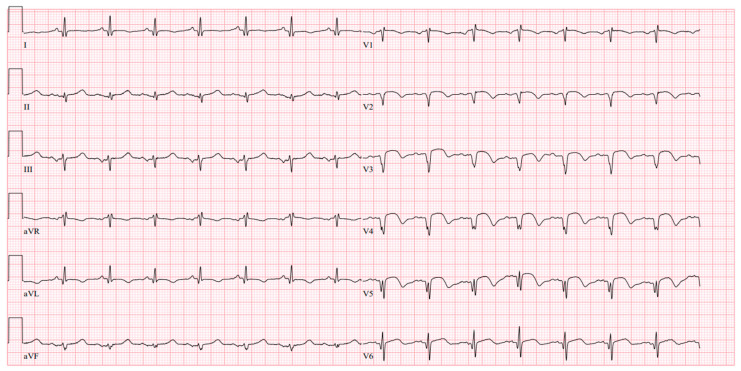
ECG performed on arrival at the PCI center.

At the PCI center, the ECG was performed, revealing a change resembling a subacute anterior MI with a left ventricular aneurysm (Figure 1).

Blood tests revealed significantly elevated levels of brain natriuretic peptide (BNP), troponin I, and D-dimer (the detailed laboratory results are shown in Table 1). The elevated C-reactive protein (CRP) level was attributed to post-MI changes, as there were no clinical signs of infection. A chest computed tomography scan was performed, showing no signs of pulmonary thromboembolism.

Prior to this event, the patient’s regular medications included antihypertensive drugs, a statin, and a proton pump inhibitor due to a history of stomach ulcers (a detailed list of medications is shown in Table 2).

**Figure 2:**
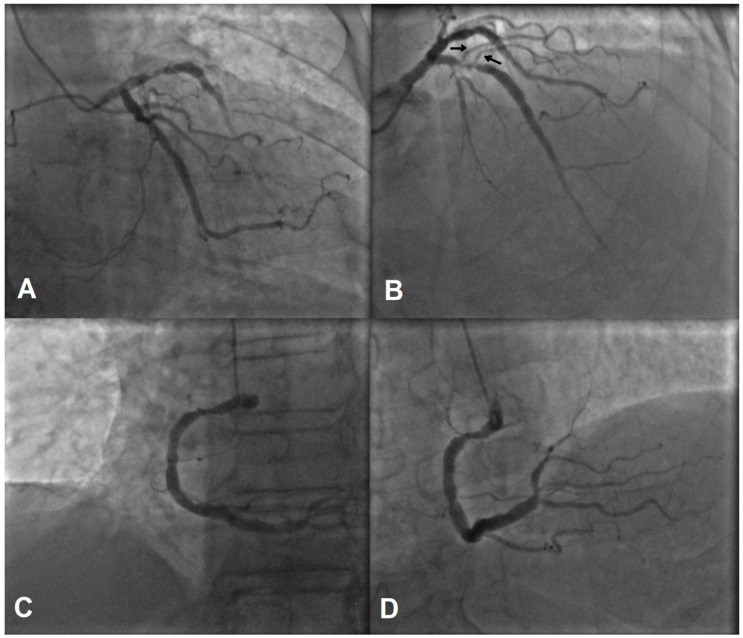
Coronary angiography. (**A**,**B**) show a subocclusion of LAD. Please note two small diagonal arteries marked with black arrows, which originate from LAD near the lesion. (**C**,**D**) show a moderate proximal RCA lesion.

Since the chest pain had occurred 10 days prior and no chest pain was present upon arrival at the PCI center, in addition to the ECG showing signs of a subacute myocardial infarction (MI), the patient was not immediately directed to coronary angiography. The angiography was performed the following day and revealed a proximal subocclusion of the left anterior descending artery (LAD), as well as moderate proximal stenosis of the right coronary artery (RCA) (see Figure 2).

**Figure 3:**
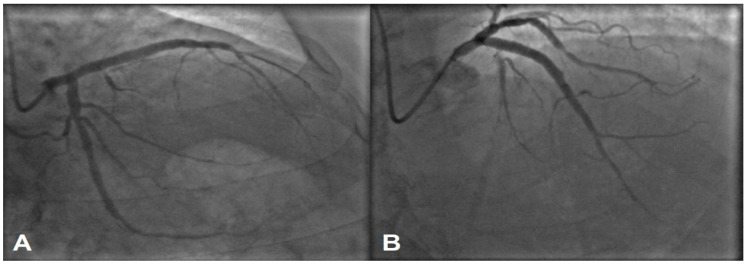
Culprit lesion was successfully stented with an optimal angiographic result (**A**,**B**). The side branches visible in Figure 2B appear to be occluded after stenting, as shown in (**B**).

The lesion was managed with a 5 × 28 mm drug-eluting stent, and an optimal angiographic result was achieved (Figure 3A). Given the large diameter of the proximal LAD, a 5 mm stent was the optimal choice after implantation. However, as shown in Figure 2, two small diagonal branches were occluded. (Figure 3B).

**Figure 4:**
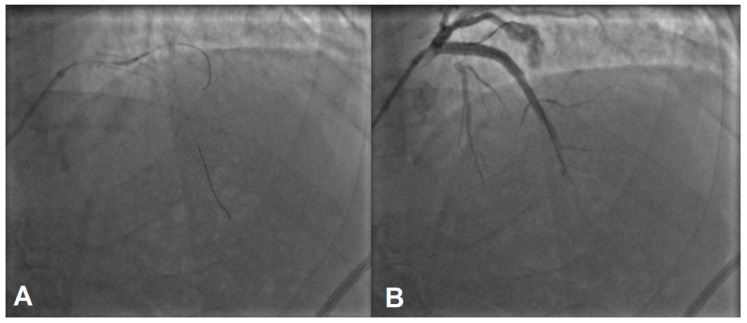
(**A**) shows rewired diagonal branch and opening of stent strut. (**B**) depicts an extravasation out of the side coronary branch.

Since the patient experienced chest pain after the stent implantation, a decision was made to try to revascularize one of the side branches. A hydrophilic wire 0.014 Light Support PT2 was introduced through the stent struts into one of the diagonal branches, and the strut was predilated with a 1.5 × 15 mm Monorail Maverick 2 balloon to restore flow to the side branch (Figure 4A). Unfortunately, the side vessel was damaged accidentally, probably with wire, leading to extravasation, as seen on the angiography (Figure 4B).

**Figure 5:**
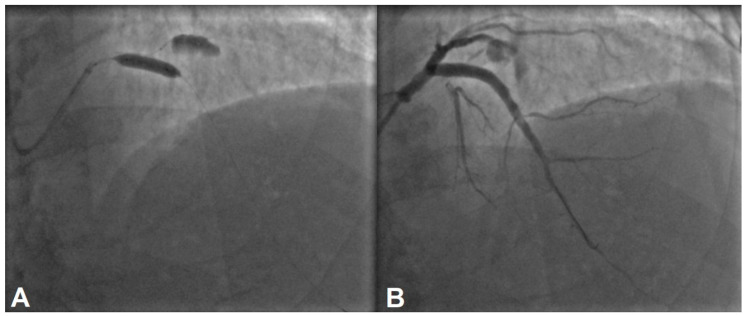
(**A**) depicts an injection of glue into the vessel; some part of the glue seems to reach a pericardium space, which causes no harm to the patient. (**B**) depicts an optimal overall result of successful LAD stenting and embolized side branch.

A balloon catheter was introduced immediately to control the extravasation into the pericardium. Since the bleeding did not cease on its own after waiting with an inflated balloon and there was no covered coronary stent of such a big diameter available at our site, an endovascular glue technique was applied. A coronary microcatheter of 1.8 French was introduced into a diagonal branch, and after flushing with glucose, a 5% injection of n-hexyl-cyanoacrylate (Magic Glue, “BALT”) and Lipiodol (1:1) solution was performed. The damaged vessel was successfully rapidly embolized, stopping the bleeding into the pericardium (Figure 5).

Echocardiography revealed a small amount of fluid, approximately 7 mm, in the pericardium, indicating a good overall result.

Control transthoracic echocardiography showed no excess pericardial fluid, indicating that the applied technique successfully managed the periprocedural extravasation complication. Overall, left ventricular contractility was not affected, and the left ventricular ejection fraction remained above 55%. The patient did not experience chest pain after the procedure and was subsequently discharged to cardiac rehabilitation on dual antiplatelet therapy with ticagrelor and aspirin.

## 3. Discussion

This case presents a rare complication of percutaneous coronary intervention: perforation of a coronary artery side branch. The perforated artery was successfully embolized using an n-hexyl-cyanoacrylate (Magic Glue, BALT) intracoronary glue injection. According to the literature, the incidence of coronary perforation during PCI ranges from 0.1% to 3%, depending on the center [12]. This complication most commonly occurs during chronic total occlusion revascularization procedures [12]. The mortality rate can be as high as 21.2%, depending on the complexity of the procedure [7]. In the vast majority of cases, perforation is detected on angiography during the procedure. However, in some instances, serial echocardiography is used to detect pericardial effusion and tamponade, leading to repeat coronary angiography [12]. Established techniques for treating such complications include covered stents, coils, coronary microspheres, thrombin injection, and surgery [13,14]. Partial damage, such as extraluminal excavation or myocardial blush without extravasation, can be managed conservatively. Severe cases with significant extravasation into the pericardium should be managed more aggressively, and serial echocardiography for the first 48 h after the procedure is strongly recommended for the early detection of cardiac tamponade and pericardial effusion [12,15]. In this case, the perforation was successfully managed using the unconventional method of intracoronary glue injection.

Goel and Syal describe the successful management of coronary artery small ramus perforation with an intracoronary injection of cyanoacrylate glue [16]. The authors used a microcatheter flushed with 5% dextrose, and the vessel was effectively occluded with 2 mL of intracoronary glue mixed with an equal part of lipiodol [16]. Rafeedheen et al. present a case of perforation following a chronic total occlusion revascularization procedure of the circumflex artery [17]. The perforated circumflex artery was successfully occluded using an intracoronary n-butyl-cyanoacrylate glue and lipiodol mix, which stopped the extravasation [17]. The authors also report an in vitro test demonstrating the recanalization of a vessel occluded by the glue, which was successful, and the glue material was found to be circumferentially present within the walls of an artificial artery—an important consideration in cases of perforation [17]. However, no such in vivo tests were performed during coronary angiography. Goel also reported a case of persistent leakage from the distal right coronary branches following RCA PCI, attributed to recurrent cardiac tamponade and drainage [18], which was managed by embolizing the distal branches with n-butyl-2-cyanoacrylate [18]. Similar cases of LAD perforation treated with cyanoacrylate glue have been reported by Trehan et al. and Mishra et al. [19,20]. Despite these reports, the use of intracoronary glue for treating coronary artery perforation is still limited in the literature, indicating a need for further research.

Intravascular cyanoacrylate glue is widely used for embolizing arteriovenous malformations in brain vessels [21], with expanded applications in peripheral vasculature [22]. It is employed to embolize tumor vessels [22,23], manage acute bleeding [22,24], and offer faster administration than micro coils [25]. This minimally invasive technique also reduces pain and recovery time compared to surgery [26], shows promise for treating lower limb varices [27], and addresses postprocedural complications like type 2 endoleak [28,29]. These studies highlight the broad applicability of glue embolization beyond heart-related vessels.

Since the perforated vessel in the presented case report originated from a large vessel 5 mm in diameter and covered stents of such a diameter were unavailable, cyanoacrylate glue looked like a feasible option at the time. Due to the small diameter, it was challenging to precisely measure the amount of glue needed, which led to some extravasation into the pericardial space, as shown in Figure 5A. There is limited evidence in the literature regarding the outcomes of such events. Eastman et al. report that household cyanoacrylate glue was successfully used to attach pericardial patches between the pericardium and lacerated myocardial tissue to stop or prevent hemorrhage [30]. They found the technique to be safe and effective, with none of the patients developing a mediastinal infection; the cyanoacrylate glue appeared to be bacterial-free and exhibited a bactericidal effect [30]. Additionally, Paez et al. found that cyanoacrylate glue was more effective than bioadhesives for bonding pericardial tissue in a calf study [31]. These studies demonstrate the effectiveness of cyanoacrylate glue for embolization, making the decision to prescribe dual antiplatelet therapy, as required for stent implantation, a safe clinical choice to manage the risk of repetitive perforated artery extravasation.

There are several cyanoacrylate glue types available for endovascular interventions. The main types include n-hexyl-cyanoacrylate, n-butyl-cyanoacrylate, and n-methyl-cyanoacrylate [22]. Additionally, a mix of n-butyl-cyanoacrylate and 2-octyl-cyanoacrylate is available, though only available in China [32]. Another available mixture is n-butyl-cyanoacrylate combined with methacrylosulpholane, which offers an extended polymerization time and reduced inflammatory reaction compared to n-butyl-cyanoacrylate alone [32]. Among the three main types mentioned, n-methyl-cyanoacrylate is the oldest; it has the fastest polymerization time, the strongest adhesive properties, and the least CH3 radical emission but appears to be the most cytotoxic and proinflammatory [22]. N-butyl-cyanoacrylate also bonds strongly with the vessel wall and has a lower cytotoxic and inflammatory effect, although it has a higher CH3 emission and slower polymerization time [22]. N-hexyl-cyanoacrylate, which was used in our case, is the newest endovascular glue among those mentioned. It has weaker adhesive properties, the slowest polymerization time, and the highest CH3 emission. However, it is the least cytotoxic and causes a milder inflammatory reaction [22]. Cyanoacrylate glue is an absorbable substance that can lead to revascularization of the occluded vessel [22]. A study by Rao shows that n-butylacrylate may be significantly absorbed after 6–20 months, which leads to revascularization in some patients [33]. The differences between the main glue types lie in the size of the molecular side chain; the larger the side chain, the longer the polymerization time, the weaker the adhesive properties, and the less severe the cytotoxic and inflammatory reactions [22,32]. Nevertheless, in our case, the time of polymerization of n-hexyl-cyanoacrylate was very fast and effective for the intervention. Unfortunately, there is still a lack of evidence regarding the absorption time for each type.

## 4. Conclusions

Coronary artery perforation is a serious complication of percutaneous coronary intervention. The most common treatment options include covered stent implantation, micro coils, and surgery. Intracoronary glue, n-hexyl-cyanoacrylate, injection, and embolization of the perforated side branch appear to be a safe, very fast, and effective technique for managing this complication. However, further research is needed to provide stronger evidence to support the routine use of this technique.

## Figures and Tables

**Table 1:** Relevant laboratory results on arrival at PCI center.

Test	Result	Laboratory Reference Range
Hemoglobin, g/L	148	125–172
Troponin I, ng/L	550 (+ *)	<35
Brain natriuretic peptide, ng/L	**1016.4 (+)**	Chronic congestive heart failure unlikely if <35 Acute heart failure unlikely if <100
D-dimer, µg/L	**860 (+)**	<250
C-reactive protein, mg/L	**57.0 (+)**	<5

* + stands for clinically significant elevated results.

**Table 2:** Medical treatment before the cardiovascular event.

Drug	Dose, Regime
Bisoprolol/Perindopril	10/5 mg o.d.
Doxazosin	4 mg o.d.
Rosuvastatin	15 mg o.d.
Pantoprazole	40 mg b.i.d.

## Data Availability

The original contributions presented in the study are included in the article, further inquiries can be directed to the corresponding author.
